# Targeting BCL-2–expressing basal-like breast cancer with BH3-mimetics

**DOI:** 10.1186/1897-4287-10-S2-A25

**Published:** 2012-04-12

**Authors:** SR Oakes, F Vaillant, E Lim, L Lee, K Breslin, F Feleppa, S Deb, ME Ritchie, E Takano, T Ward, SB Fox, D Generali, GK Smyth, A Strasser, DCS Huang, JE Visvader, GJ Lindeman

**Affiliations:** 1 The Walter and Eliza Hall Institute of Medical Research, Parkville VIC 3052, Australia; 2 The University of Melbourne, Parkville, VIC 3010, Australia; 3 The Royal Melbourne Hospital, Parkville VIC 3050, Australia; 4 Peter MacCallum Cancer Centre, East Melbourne, VIC 3002, Australia; 5 Ospitalieri di Cremona, Cremona 26100, Italy

## 

Impairment of apoptosis is a hallmark of cancer and can result in resistance to chemotherapy. Tumour resistance to apoptosis is frequently acquired through deregulated expression of BCL-2 family members or inactivation of the p53 tumour suppressor pathway. Over-expression of the pro-survival protein BCL-2 is common in breast cancer (where it is readily detected by immunostaining), and has been shown to be an important prognostic marker. A potential role for BCL-2 as a *therapeutic target* in breast cancer, however, has not been explored. Recently, small molecules termed ‘BH3-mimetics’ have been developed that mimic the action of pro-apoptotic BH3-only proteins. These bind and neutralize pro-survival proteins including BCL-2.

Tissue microarrays containing 197 primary breast tumours were evaluated for the expression of BCL-2, its anti-apoptotic relatives MCL-1 and BCL-XL, and the pro-apoptotic BH3-only ligand BIM. These proteins were co-expressed at relatively high levels in a substantial proportion of heterogeneous breast tumours, including clinically aggressive basal-like cancers. To determine whether the BH3-mimetic ABT-737 that neutralizes BCL-2, BCL-XL and BCL-W, had potential efficacy in targeting BCL-2-expressing basal-like triple negative tumours, we generated a panel of primary breast tumour xenografts in immunocompromised mice and treated recipients with either ABT-737, docetaxel or a combination. Tumour response and overall survival were significantly improved by combination therapy, but only for tumour xenografts that expressed elevated levels of BCL-2. Treatment with ABT-737 alone was ineffective, suggesting that ABT-737 sensitizes the tumour cells to docetaxel. Combination therapy was accompanied by a marked increase in apoptosis and dissociation of BIM from BCL-2. Notably, BH3-mimetics also appeared effective in BCL-2-expressing xenograft lines that harbored p53 mutations.

In summary, primary breast tumour xenograft models that recapitulate the phenotype of the primary tumour have been developed as useful ‘proof-of-principle’, pre-clinical models. Our findings provide the first *in vivo* evidence that BH3-mimetics can be used to sensitize primary BCL-2-expressing breast tumours to taxane chemotherapy. Our results suggest that elevated BCL-2 expression constitutes a predictive response marker in breast cancer. These findings provide a rationale for the development of clinical protocols using the oral analogue ABT-263 (navitoclax) as an adjunct to taxane chemotherapy in BCL-2-expressing basal-like and luminal breast cancer.

